# Biomarkers in Hepatobiliary Cancers: What Is Useful in Clinical Practice?

**DOI:** 10.3390/cancers13112708

**Published:** 2021-05-30

**Authors:** Alice Boilève, Marc Hilmi, Matthieu Delaye, Annemilaï Tijeras-Raballand, Cindy Neuzillet

**Affiliations:** 1 Gustave Roussy, Département de Médecine Oncologique, 94805 Villejuif, France; alice.boileve@gustaveroussy.fr; 2 GERCOR Group, 151 rue du Faubourg Saint-Antoine, 75011 Paris, France; hilmi.marc@gmail.com (M.H.); mdelaye@sfr.fr (M.D.); araballand@oncomega.com (A.T.-R.); 3 Département de Médecine Oncologique, Curie Institute, 92210 Saint-Cloud, France; 4 OncoMEGA, 75010 Paris, France

**Keywords:** hepatocellular carcinoma, biliary tract cancers, cholangiocarcinoma, immune checkpoint inhibitor, targeted therapy, biomarker

## Abstract

**Simple Summary:**

In oncology, a new era has emerged in the last ten years with the development of targeted and immune therapies. In hepatocellular carcinoma (HCC), several targeted agents (sorafenib, lenvatinib, cabozantinib, regorafenib, and ramucirumab) are approved and immunotherapy is now validated in combination with bevacizumab, while theragnostic biomarkers are lacking for patient selection. Conversely, in biliary tract cancer (BTC), immune therapies are still investigational while targeted therapies are now crucial considering the complex molecular landscape of BTC. In this review, we provide an overview of (i) the main prognostic biomarkers in HCC and BTC, (ii) the main theragnostic biomarkers in both tumors, and lastly (iii) what is recommended in clinical practice.

**Abstract:**

Hepatocellular carcinoma (HCC) and biliary tract cancers (BTC) exhibit a poor prognosis with 5-year overall survival rates around 15%, all stages combined. Most of these primary liver malignancies are metastatic at diagnostic, with only limited therapeutic options, relying mainly on systemic therapies. Treatment modalities are different yet partially overlapping between HCC and BTC. The complex molecular profile of BTC yields to several actionable therapeutic targets, contrary to HCC that remains the field of antiangiogenic drugs in non-molecularly selected patients. Immunotherapy is now validated in the first line in HCC in combination with bevacizumab, while clinical activity of single agent immunotherapy appears limited to a subset of patients in BTC, still poorly characterized, and combinations are currently under investigation. In this review, we provide a critical evaluation and grading of clinical relevance on (i) the main prognostic biomarkers in HCC and BTC, (ii) the main theragnostic biomarkers in both tumors, and lastly (iii) what is recommended in clinical practice.

## 1. Introduction

Hepatocellular carcinoma (HCC) is the third leading cause of cancer death worldwide [1], and the most frequent primary liver cancer (65,000 and 42,000 new cases/per year in Europe and the United States, respectively). In most cases, HCC arise on underlying chronic liver diseases, mainly due to chronic infections by hepatitis B virus (HBV) or hepatitis C virus (HCV), alcohol, or fatty liver disease [2,3,4,5,6].

Biliary tract cancers (BTCs) develop from the epithelium of bile ducts, gallbladder, or ampulla of Vater. BTCs encompass a heterogeneous group of tumors with specific anatomical, biological, prognostic, and therapeutic features [7,8,9]. BTCs comprise of gallbladder carcinoma (GBC) and cholangiocarcinoma (CCA). CCA are divided into intrahepatic CCA (iCCA) and extrahepatic CCA (eCCA) themselves subdivided into distal and perihilar CCA [7,10]. Regardless of the disease stage, GBC and CCA represent 65% and 35% (15% for iCCA and 25% for eCCA) of BTCs, respectively [10]. BTC is a rare cancer in Western countries (>6/100,000), and represent approximately 3% of all gastrointestinal malignancies [11]. Yet it is the most frequent hepatobiliary cancer after HCC [12]. Moreover, the overall incidence of CCA is rising, mostly due to chronic liver diseases (mainly fatty liver disease related to metabolic syndrome) [13,14]. A global heterogeneity of incidence is observed, with more cases in South-East Asia [15] (due to fluke infections of the liver and the biliary tree). In Europe and the United States, the majority of cases are sporadic. Main risk factors are described as liver cirrhosis, intrahepatic biliary stone disease, primary sclerosing cholangitis, biliary malformations, and rarely genetic syndromes such as Lynch syndrome [16,17].

Surgery is the cornerstone curative treatment of BTC and HCC. Depending on HCC stages, liver transplantation, surgical resection, or radiofrequency ablation can be discussed as curative intent treatment while intra-arterial liver therapies and systemic therapies are palliative [18]. In patients with HCC limited to the liver and sufficient liver function, resection, and local ablation are the recommended curative locoregional therapies [19]. Nevertheless, recurrence rates are high, and liver transplantation remains superior in terms of disease control and long-term survival. Nevertheless, this treatment is limited because of limited available donor organs. Transarterial chemoembolization can be used to bridge patients to transplantation but also as a standard of care for patients not suitable for other local therapies.

Liver transplantation in BTC remains controversial [20,21] but might be proposed as part of a multimodal protocol (Mayo Clinic) for selected patients with unresectable perihilar CCA without metastatic disease. Radiofrequency ablation and stereotactic radiotherapy might also be an option for non-operative patients with small iCCA [22]. Forty percent of HCC are diagnosed at advanced stages. Five-year OS is around 15%, all stages taken together [18]. Regarding BTC, only one third of cases of them are resectable at diagnosis, and 5-year overall survival (OS) regardless the stage is as low as 5–15% [10,23]. For resected perihilar CCA and iCCA, a 5-year OS of about 30% has been reported [24,25,26].

**Figure 1:**
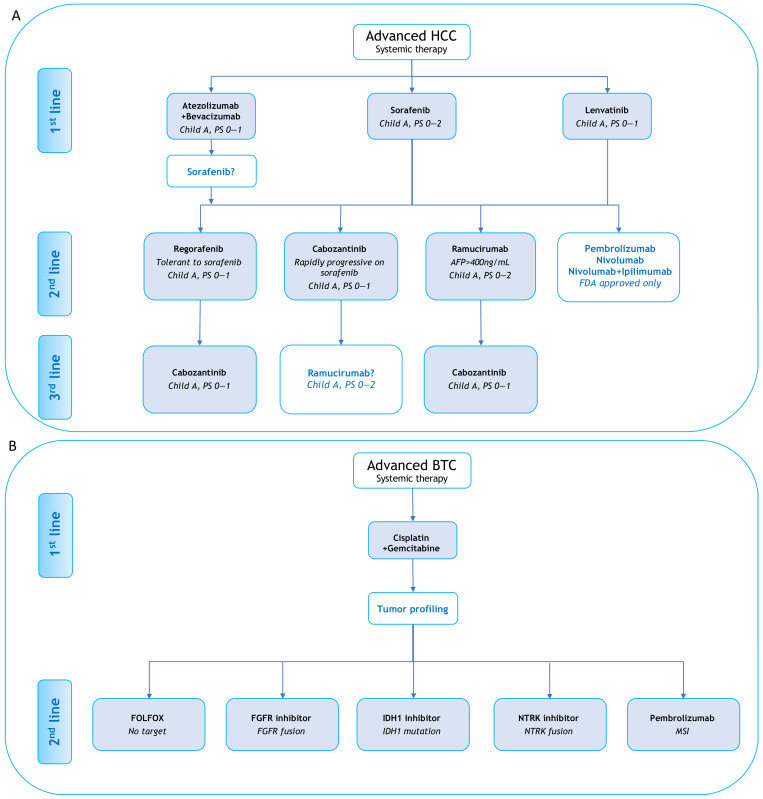
Systemic treatments for advanced hepatocellular carcinoma (**A**) and biliary tract cancers (**B**).

In case of unresectable or metastatic disease, systemic therapies are indicated [18,27] (Figure 1). In HCC, only angiogenesis inhibitors were approved until recently with sorafenib and lenvatinib approved for the first-line treatment of advanced HCC patients [28,29]. Cabozantinib, regorafenib, and ramucirumab are also therapeutic options in pre-treated patients. No predictive biomarkers are used yet to select patients for antiangiogenic therapy in HCC with the exception of AFP level for ramucirumab. For BTC, first-line standard chemotherapy is cisplatine-gemcitabine regimen with a median OS of 11.7 months [30] and in second line, a combination of 5-fluorouracil and oxaliplatine can be proposed [31]. The poor results obtained with systemic chemotherapy in BTC stress a need for efficient targeted therapies and the need for molecular profiling. In the past years, next-generation sequencing (NGS) revealed a complex molecular background in BTC with potential clinical implication [32]. Until recently, no targeted therapy was approved for BTC, until the Food and Drug Administration (FDA) and the European Medicines Agency (EMA) approved pemigatinib in 2020 for CCA with an fibroblast growth factor receptor 2 *(FGFR2*) rearrangement or fusion [33] and ivosidenib in case of isocitrate dehydrogenase 1 *(IDH1)* mutation [34] for FDA.

Recently, immunotherapy was validated in first-line of HCC, with the combination of atezolizumab and bevacizumab [35]. FDA had also approved nivolumab, nivolumab plus ipilimumab, and pembrolizumab in the second line (Figure 1). To date, in BTC, clinical activity of single agent immunotherapy appears limited to a subset of patients, still poorly characterized, and combination are currently under investigation. The only FDA approval of immunotherapy for BTC is restricted for microsatellite instability (MSI) tumor.

Interestingly, HCC and BTC share a common unique microenvironment and immune niche, i.e., the liver, which is a physiologically immunotolerant organ. HCC are immunogenic but immunosuppressed and highly vascularized tumors with no approved targeted therapies except from angiogenesis inhibitors, while BTCs are an anatomically, molecularly, and therapeutically heterogeneous group of tumors with a promising use of dedicated targeted therapies.

**Figure 2:**
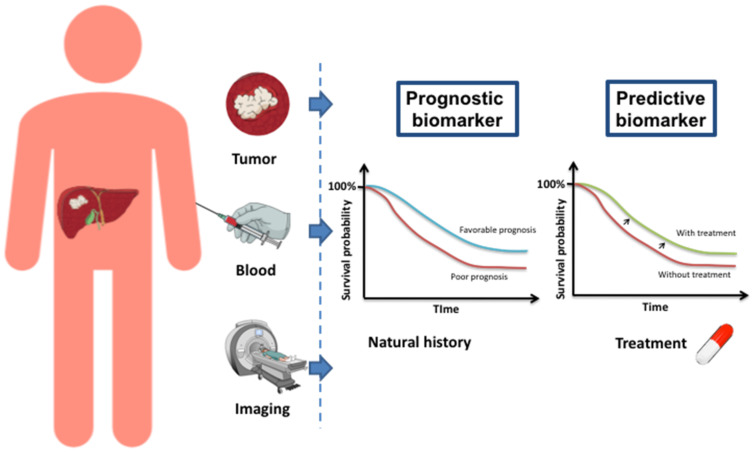
The difference between a prognostic and a predictive biomarker.

Theragnostic biomarkers, with a potential therapeutic impact, are developed in both tumors with different approaches. A biomarker can be defined as “a characteristic that is measured as an indicator of normal biological processes, pathogenic processes or responses to an exposure or intervention” [36]. Biomarkers have applications in several clinical areas and can be very diverse in nature: clinical, molecular, or imaging (Figure 2). In cancer biology, four types of biomarkers are distinguished: (i) diagnostic biomarkers, which help in the diagnosis or identification of subclasses of a particular disease; (ii) prognostic biomarkers, which are associated with a more or less favorable disease course in terms of progression-free and/or overall survival, irrespective of the treatments administered (“natural history”); (iii) predictive biomarkers, which predict the activity of a specific treatment, used as a tool for therapeutic decision-making; (iv) diagnostic companion biomarkers, used to define a subgroup of patients for whom a given treatment has been shown to be effective and for which a therapeutic indication is reserved.

Therefore, identification of biomarkers is crucial to improve patients’ management and outcome. Extensive research is ongoing to help in diagnosis and to predict prognosis and response to treatment of patients with HCC and BTC. These two tumors share common biological features. Yet HCC is a tumor for which biomarkers are lacking, while in BTC a lot of biomarkers have been identified, some of them with a clinical impact for patients’ management but not all of them are ready for the routine clinical practice. In this review, we provide an overview of (i) the main prognostic biomarkers in HCC and BTC, (ii) the main theragnostic biomarkers (predictive and diagnostic companion) in both tumors, and lastly (iii) what is really useful in clinical practice. We therefore propose a critical evaluation and grading of clinical relevance of existing biomarkers in HCC and BTC.

## 2. Main Prognostic Biomarkers in Hepatocellular Carcinoma and in Biliary Tract Cancer

### 2.1. In Hepatocellular Carcinoma

Different prognostic biomarkers were proposed in HCC based on clinical, biological, or molecular data. Extensive programs from large randomized clinical trials in HCC were launched to investigate the prognostic value of identified biomarkers.

#### 2.1.1. Classical Biomarkers

Clinical markers have long been the keystone biomarkers in HCC, such as extra liver extension or macrovascular invasion that are associated with a poor prognostic [37].

Data from the SHARP trial alone evaluating sorafenib as front-line in HCC revealed that vascular invasion and high levels of specific proteins such as angiopoietin 2 (Ang2), alpha-feto protein (AFP), and vascular endothelial growth factor (VEGF) were independent predictors of poor OS [38]. Since then, the AFP prognostic value has been confirmed in several studies [39]. By pooling the SHARP and ASIA-PACIFIC trials, Bruix et al. suggested that patients treated with sorafenib had worse OS when they had non-HCV related HCC (*p* = 0.02) [40]. 

Other studies suggested that inflammation-based cancer-prognostic biomarkers had an independent negative prognostic value such as the neutrophil-to-lymphocyte ratio (NLR) [41,42,43], the systemic immune-inflammation index (SII) (4), and the Glasgow Prognostic Score (GPS) [44]. In addition, sarcopenia is associated with cancer-related cachexia and systemic inflammation and has been described as a strong negative prognostic indicator for many cancer types [45]. In HCC, several studies confirmed the prognostic value of sarcopenia in the localized [46,47,48,49] and advanced settings [48,50].

The emergence of NGS paved the way for new and personalized biomarkers. Therefore, in the last few years, new biomarkers have emerged, such as immune or molecular biomarkers. 

#### 2.1.2. Emerging Biomarkers

The type of immune cells within the tumor microenvironment has been described as a prognostic marker in HCC. On the one hand, an enrichment in the immunosuppressive T regulatory lymphocytes (T cells) and myeloid-derived suppressor cells (MDSC) is linked to a poorer prognosis, whereas on the other hand cytotoxic T cells, B cells, NK cells, and dendritic cells are associated with an improved prognosis [51,52].

Another study used gene-expression in the tumor microenvironment of HCC and defined two major subtypes. The first subtype was associated with an increased expression of immune-related genes and of worse prognosis than the second subtype [53]. Similarly, Chen al. classified HCC tumors into two categories based on the signature of 18 immune-associated genes with opposite prognosis values [54], and described a specific seven-biomarkers signature based on five immunity- and two ferroptosis-related genes expression as an independent predictive factor of OS [55]. 

Furthermore, programmed-death ligand 1 (PD-L1), the ligand of the immune checkpoint receptor programmed-death 1 (PD-1), is expressed on the membrane of tumor cells and in the peritumoral stroma in HCC, and is associated with poor prognosis [56,57]. Calderaro et al. studied 217 immunotherapy-naïve, resected HCCs and found that 75% of patients expressed PD-L1 in tumor cells with a wide range of intensity. Markers of tumor aggressiveness such as poor differentiation, vascular invasion, and high AFP levels were associated with PD-L1 expression [58]. Another study found the same poor prognostic value of PD-L1 independently of other known clinicopathological prognostic factors in HCC. Similarly, a high PD-L1 expression in patient serum (measured by enzyme-linked immunosorbent assay (ELISA)) or in peripheral blood mononuclear cells (measured by flow cytometric analysis) seems associated with worse outcomes in HCC [59].

Several studies used molecular data to discover prognostic biomarkers in HCC due to the high heterogeneity of this tumor type. A recent study from the international consortium “The Cancer Genome Atlas (TCGA) Research Network” has generated a molecular classification of the HCC microenvironment. Several integrated clusters (iClust) with different prognostic values based on the analysis of 363 cases by whole-exome sequencing and DNA copy number analysis, and the additional analysis of 196 cases for DNA methylation, RNA expression, micro-RNA (miRNA), and proteomics has been identified [60]. Patients with iClust 1 had the worst prognosis with high-grade tumors (overexpression of proliferation markers) and macrovascular invasion (overexpression of angiogenic genes). iClust 1 was associated with younger age, Asian ethnicity, female gender, a low mutation frequency of *CTNNB1*, a low expression of TERT, and an epigenetic silencing of cyclin-dependent kinase inhibitor 2A (*CDKN2A*), while iClust 3 (30%) was inflammatory (high chromosomal instability) and had the best survival. *CTNNB1* codes for beta-catenin, a dual function protein involved in cell–cell adhesion and gene transcription. TERT (telomerase reverse transcriptase) is the catalytic subunit of the enzyme telomerase. CDKN2A, also known as cyclin-dependent kinase inhibitor 2A is a regulator of cell cycle. The dominant subset was iClust 4 (depleted in lymphocytes, 40%) but showed no prognostic value. As compared to other clusters, iClust 2 and iClust 4 had higher CD8/Treg ratios and were enriched in highly immunogenic peptides generated from non-silent coding mutations in the cancer cell genome, also called neoantigens. 

Hoshida et al. studied transcriptomic data from eight independent cohorts representing 603 patients with HCC and found three robust classes with separate prognostic values [61]. Two poor prognosis subclasses (subclass 1 and subclass 2) were associated with activations of the WNT signaling pathway and the proliferation pathway (MYC and AKT activations), respectively, whereas the third good prognosis subclass (sublcass 3) was associated with a hepatocyte-like phenotype.

Boyault et al. deciphered HCC tumors based on transcriptomic data and found similar results by isolating one group with beta-catenin mutations and WNT pathway activation whereas another group showed an activation of the cycle [62]. In these last two studies, poor prognostic molecular groups were associated with low differentiation and vice versa for good prognostic molecular groups indicating an accurate correlation between molecular and histological data [63,64]. The negative prognostic value of the cell division cycle-associated genes overexpression was suggested in another study [65]. 

Other minimally invasive molecular parameters available in the bloodstream could be prognostic in HCC. Indeed, biomarkers such as the amount of circulating tumor cells and circulating nucleic acids can reflect the tumor size, vascular invasion, and metastases, or the proliferation rate by studying mutations and hypermethylation of specific genes in circulating DNA [66,67]. Circulating transcriptomic data also give prognostic information in HCC through oncogenic miRNAs such as miR-21 [68]. 

### 2.2. In Biliary Tract Cancers

#### 2.2.1. Classical Biomarkers

Commonly admitted prognostic factors in BTCs are mostly clinical or based on pathological characteristics as TNM classification for resectable tumors [69], particularly lymph node invasion [70,71], resections margins [72], histological subtype [73], and performance status, prior to resection of primary tumor and peritoneal carcinomatosis at advanced stages [74].

However, other biomarkers have been established for years. Carcinoembryonic antigen (CEA) and Antigene Carbohydrate 19-9 (CA 19-9) are key biomarkers to monitor BTCs and evaluate prognosis of patients. Prognostic value of these biomarkers has been assessed in resectable BTCs. In two series of 106 and 168 patients with resected BTCs, high preoperative CA 19-9 expression was associated with poor outcomes [75,76]. For the first study [75], preoperative CA 19-9 (≥200 IU/mL) was associated with poor outcome with a hazard ratio (HR) of 2.17 (95% CI: 1.04–4.43) and postoperative CA 19-9 (≥37 IU/mL) with HR = 7.46 (95% CI: 3.64–15.72). For the second study [76], preoperative CA 19-9 (≥150 IU/mL) was associated with poor outcome with HR = 2.23 (95% CI: 1.14–4.40). In another series of 190 patients, preoperative CEA (with a cut-off value of 4.55 µg/L, HR = 1.030 (95% CI: 1.002–1.058)), but not CA 19-9, was associated with prognosis after tumor resection [77]. Nevertheless, these results should be tempered by the relatively small size of these studies with some non-uniform results, particularly in term of cut-off values, which differ between studies. At advanced stages, pretreatment CA 19-9 levels and CA 19-9 decrease after chemotherapy are of prognostic relevance in patients with BTC [78]. Importantly, it should be noted that these biomarkers are unspecific, particularly CA 19-9, which can be elevated in several clinical situations as decompensated diabetes [79] or cholestasis (frequently observed in patients with advanced disease) [80], which makes it difficult to interpret an elevated marker [81].

More than the absolute value of these biomarkers, it is rather the kinetics under or after treatment that would be prognostic. This was evaluated in patients with advanced BTCs who were treated with gemcitabine-based chemotherapy [82]. In this cohort of 179 patients, a decrease ≥50% in CA 19-9 was associated with better survival (16 versus 9 months). This was not found with CEA. Of note, in this study, the absolute CA 19-9 level was also found to be a predictive factor. Importantly, it should be noted that these biomarkers are unspecific, particularly CA 19-9, which can be elevated in several clinical situations as decompensated diabetes [79] or cholestasis [80], which makes it difficult to interpret an elevated marker [81]. 

Cytokeratin-19 fragment (CYFRA 21-1) has also been studied as prognostic biomarkers and its elevated preoperative dosage in patients with operable iCCAs shows interesting results [83]. The optimal cut-off value for CYFRA 21-1 was 2.7 ng/mL (HR = 2.9 (95% CI: 1.1–7.8) for OS and HR = 6.0 (95% CI: 1.8–20.0)).

#### 2.2.2. Emerging Biomarkers

##### Molecular Biomarkers

NGS has identified several molecular signatures, which can be used as prognostic biomarkers. In 2016, Javle and al. performed hybrid capture-based comprehensive genomic profiling on 554 BTC, including 412 iCCAs and 57 eCCAs [84]. Several gene alterations were observed with differences between iCCAs and eCCAs. In iCCA, the most frequent alterations were tumor protein 53 (TP53), CDKN2A/B, V-Ki-ras2 Kirsten rat sarcoma viral oncogene homolog (KRAS), AT-rich interactive domain-containing protein 1 (*ARID1*), and IDH in 27%, 27%, 22%, 18%, and 16% of tumors respectively. IDH1 mutation and FGFR translocations were almost exclusively found in iCCAs. In eCCAs, the most frequent alterations were *KRAS*, *TP53*, *SMAD4,* and *CDKN2A* in 42%, 40%, 21%, and 17% of tumors respectively. TP53 and KRAS mutations were associated with a shorter OS in the iCCA population, consistent with other observations in large cohort studies [85,86]. *FGFR2* translocations were associated with better OS, while IDH1/IDH2 mutations were not associated with prognosis. 

In more recent work, Lowery et al. analyzed tumors of 195 patients using a whole exome plus selected introns-sequencing assay (152 iCCAs and 43 eCCAs) [87]. In this study, the most frequent mutations in iCCAs were *IDH1*, *ARID1A*, *BAP1*, *TP53,* and *FGRF2* in 30%, 23%, 20%, 20%, and 14% of cases respectively. CDKN2A/B and Receptor tyrosine-protein kinase erbB-2 (ERBB2) were pejorative prognostic biomarkers. These results are consistent with previous studies except for frequency of gene alterations.

Other studies identify epidermal growth factor receptor (EGFR) mutation [88,89,90,91] and PD1-PDL1 expression [92] as potential biomarkers of worst prognosis. The prognostic value of *IDH1/2* remains unclear, since some studies found an inverse correlation between *IDH1* mutation and survival [93] whereas some other studies found none [84,94].

A prognostic model for iCCAs was developed, combining these molecular alterations with clinical and pathological criteria [95], but still need to be confirmed for clinical practice.

##### Immune Microenvironment

The role of the immune microenvironment in tumor progression and therapy resistance is well established in BTC [39], but its specific role still needs to be clarified. According to TCGA, 70% of BTC are enriched in immune cells, and 30% are depleted in lymphocytes, with a balanced macrophages-to-lymphocytes ratio [41]. Moreover, the prognostic value differs depending on the type of immune infiltrate. Indeed, natural killer lymphocytes, CD8-positive lymphocytes, and major histocompatibility complex class I (MHCI) expression are associated with a prolonged survival [26,27,28]. On the contrary, neutrophils and M2-macrophages are associated with poor survival. Tregs showed inconsistent prognostic value [42,43]. Moreover, 10–30% of BTCs tumor cells express PD-L1 [44] with a higher density of tumor-infiltrating lymphocytes (TILs), which is associated with a better response to immune checkpoint inhibitors (ICIs) [45].

Moreover, potential candidates for ICI were identified by molecular studies. Four subtypes of BTCs according to the gene expression of 260 BTCs were described [26]. In Cluster 4 (40% of patients), there was a higher mutation load and higher expression of immune checkpoint genes (CTLA4, LAG3, TNFRSF9, PDCD1, BTLA, IDO1, HAVCR2, and *TNFRSF4*). The prognosis of this good candidate subgroup to ICI was poor. Conversely, based on the fluke status, Jusakul et al. defined also four subtypes of BTCs [23]. Cluster 3, including iCCA mostly fluke-negative, overexpressed immune checkpoint genes (*PD-1, PD-L2*, and *BTLA*). These immunogenic iCCA were mutually exclusive with Cluster 4 (IDH/FGFR driven iCCA).

#### 2.2.3. Circulating Tumor Cells

More recently, circulating tumor cells (CTCs) have been described to be a tool for diagnosis and prognosis of BTCs, and in other cancers. CTCs can be identified by several methods (e.g., real time PCR, immunocytochemistry, and flow cytometry), which are not all approved and used in current practice yet. First description of CTCs in BTCs was made in 2012 in a small series [96], with CTCs identified in 4 of 16 patients. The presence of CTCs was associated with worse outcomes. 

This result was confirmed in a larger series of 88 patients in which a CTCs count over 2/7.5 mL was associated with tumor extend and inversely correlated with survival [97]. The reasons of this correlation are not resolved yet but part of the answer can be found in the work of Arnoletti et al., which showed that CTCs of pancreatic cancers and BTCs are in constant interaction with immune system, conferring a relative resistant to T lymphocytes cytotoxic activity and apoptosis and a capacity of proliferation and growth.

Other circulating biomarkers have been developed for non-invasive diagnosis of BTCs (for example extracellular vesicles, ctDNA, and ctRNA), and could be useful, especially their kinetics, to monitor and evaluate the prognosis of these patients. Indeed, they are an indirect witness of tumor cell proliferation and differentiation (cell-free non-coding mRNA) [98,99] or of immune reaction (IL-6) [100,101].

## 3. Predictive Markers of Treatment Response

The identification of predictive biomarkers of response to systemic therapies has become a major issue in the era of personalized medicine. Predictive biomarkers are intended to be used as therapeutic decision support tools and are often explored in large clinical trials in order to select patient populations that will best respond to treatment. When they are used to select treatment options for patients, they are referred to as “companion diagnostics” [102].

### 3.1. In Hepatocellular Carcinoma

The choice of treatment for patients with HCC is based on the performance status (PS) (European Collaborative Oncology Group (ECOG) score), the underlying liver function (Child–Pugh score), and the tumor burden according to the “Barcelona Clinic Liver Cancer” (BCLC) classification [18,37]. Untreated patients with advanced HCC, ECOG PS 0–2, and a preserved liver function should be treated with systemic therapies such as anti-angiogenics alone (sorafenib and lenvatinib) or in combination with immune therapies (atezolizumab and bevacizumab). Other antiangiogenics such as cabozantinib, ramucirumab, and regorafenib are therapeutic options for second line and beyond. 

#### 3.1.1. Angiogenesis Inhibitors

Following the two pivotal phase 3 trials SHARP [28] and ASIA-PACIFIC [103], sorafenib became the standard first-line treatment for advanced HCC in 2009. No predictive markers had been identified in the ancillary analyses from the SHARP trial alone [38] but by pooling the SHARP and ASIA-PACIFIC trials, the benefit of sorafenib was greater in patients without extrahepatic spread, with HCV and a low NLR [40]. In addition, several predictive biomarkers for sorafenib response have been proposed in clinical studies such as Ang2 [104], miRNAs [105,106,107], phosphorylated ERK, or VEGFR-2 [108], alterations of the mechanistic target of rapamycin (mTOR) signaling pathway [109], amplifications of fibroblast growth factor 3/4 (FGF3/4) [110], or VEGFA(44), polymorphisms of VEGF [111] and imaging criteria [112]. A statistical model was also built to predict survival in patients undergoing sorafenib treatment based on baseline clinical features such as vascular invasion, age, ECOG score, AFP, albumin, creatinine, aspartate aminotransferase, extra-hepatic spread, and aetiology [113]. Similarly, a higher prognostic nutritional index based on albuminemia and lymphocyte count was positively associated with OS in sorafenib-treated patients [114]. However, none of these biomarkers has been validated and their use is not allowed in clinical practice yet.

Furthermore, since the approval of lenvatinib as front-line treatment of HCC following the REFLECT trial [29], studies assessed biomarkers promoting the use of lenvatinib or sorafenib. One ancillary study of the REFLECT trial suggested that having higher VEGF- and FGF-family gene-expression levels was associated with better OS in the lenvatinib arm [115] and a meta-analysis highlighted that lenvatinib could be more efficient in hepatitis B virus (HBV) patients than sorafenib [116]. Nonetheless, the current selection between sorafenib or lenvatinib is not based on clinical or biological markers but rather on the toxicity profile.

Regarding the second line in advanced HCC, the REACH-1 phase 3 trial, evaluating ramucirumab, an anti-VEGFR-2 monoclonal antibody, did not met the primary objective in the overall patient population. However, a benefit was shown in the subgroup of patients with high AFP levels (≥400 ng/mL) before treatment [117]. The REACH-2 phase 3 trial, using the same design with an exclusive inclusion of patients with AFP levels above 400 ng/mL, showed a significant modest survival benefit as compared to placebo (8.5 months versus 7.3 months, HR = 0.71, *p* = 0.019) [118]. Thus, AFP was shown to be both a prognostic and a predictive biomarker, with the result of ramucirumab being the first biomarker-guided therapy in HCC. 

Teufel et al. analyzed samples from the RESORCE trial that led to approval of regorafenib in the second line setting and found that five proteins and nine miRNAs were significantly associated with regorafenib response [119]. However, no biomarker has been identified to guide the choice between the three recommended angiogenesis inhibitors (cabozantinib, ramucirumab, and regorafenib) in patients with pretreated HCC. In addition, the iClust 1, corresponding to a wound healing profile (10%) from the TCGA analysis [60], suggested that antiangiogenics may be useful in this small group since their tumor had a higher expression of angiogenic genes. 

Functional imaging has also been developed as a predictive biomarker of response to antiangiogenic drugs. First, dynamic contrast-enhanced magnetic resonance imaging (DCE-MRI) measures changes in tumor blood flow, vascular permeability, and intravascular and interstitial volumes. In a study evaluating sorafenib vs. tegafur-uracil in advanced HCC, DCE-MRI was performed before treatment and after 14 days of treatment [118]. They measured the volume transfer constant, called Ktrans, and showed that Ktrans was higher in patients with disease control compared to progressive disease. A vascular response (≥40% Ktrans decreased after treatment) was associated with a better PFS and OS. Positron emission tomography (PET) with 18F-fluorodeoxyglucose (FDG) can be used as a surrogate of viable tumors. FDG-PET can help to predict prognosis in HCC patients receiving hepatic arterial infusion of chemotherapy or transcatheter arterial chemoembolization, but could also help to predict efficacy of sorafenib [120].

Finally, few oncogenic drivers were identified as actionable targets for therapy in HCC and does not yet allow personalized treatments in clinical practice [121]. Overall, there is no currently available validated predictive biomarker for routine practice to select patients for targeted therapies in advanced HCC. Identification of predictive biomarkers for targeted therapies such as sorafenib could help clinicians in the daily management of these patients, mostly in light of the new therapeutic options available in the first line [122].

#### 3.1.2. Immunotherapies

Several clinical trials evaluated ICI in patients with advanced HCC and searched for predictive biomarkers. The CHECKMATE-040 phase 1/2 trial evaluated nivolumab in both treatment-naive and previously sorafenib-treated patients. No significant difference of the overall response rate (ORR) according to treatment exposure or viral infection was shown [123]. Similarly, the analysis of the KEYNOTE-224 phase 2 trial evaluating pembrolizumab in the second line did not find any predictive value of biomarkers such as age, viral or non-viral etiology, AFP levels, BCLC stage, macrovascular invasion, extrahepatic metastases, and tumoral PD-L1 expression [124]. Nonetheless, the combined positive score (CPS) defined by the number of PD-L1+ cells (≥1%) (tumor cells, lymphocytes, and macrophages) divided by the total number of tumor cells was significantly associated with better ORR (32% versus 20%, *p* = 0.021) and PFS (*p* = 0.026). 

Recently, the IMbrave150 phase 3 trial showed superiority of the combination of bevacizumab and atezolizumab versus sorafenib in the first line setting of HCC in terms of OS (HR = 0.59, *p* < 0.001) [35], setting the combination of ICI and angiogenesis inhibitors as a new standard of care. In this study, the predictive value of ethnicity, macrovascular invasion, extrahepatic spread, PD-L1 status, and baseline AFP level (<400 vs. ≥400 ng/mL) is not yet described. Similarly, early phase studies assessing combination of ICIs such as nivolumab plus ipilimumab [125], and durvalumab plus tremelimumab [126] showed interesting ORR despite high rates of grade 3/4 adverse events leading in 5–7.5% of discontinuation. Thus, guiding patient selection based on biomarkers will be mandatory for ICI combination and are planned to be explored in larger randomized clinical trials (NCT02519348, NCT03298451, and NCT01658878).

In addition to clinical trials, other studies have suggested potential biomarkers for immunotherapies. In a cohort of 956 HCC samples, an IFN-pathway signature was displayed in half of the 25% lymphocytes-rich tumors [127], which has been described to be predictive of ICI efficacy in other solid tumors [128]. A second study explored the tumor immunological microenvironment from the TCGA and GEO databases and created an index to quantify the infiltration pattern predictor of immunotherapy response [129]. In a complementary manner, a prospective study in 31 patients with HCC treated by ICI showed that WNT/β-catenin pathway alterations are associated with less disease control rate (DCR) (0 versus 53%) and worst OS (9.1 versus 15.2 months) [130]. Moreover, the use of markers of genomic instability such as MSI, high tumor mutational burden (TMB) or *POLE* mutations does not seem relevant for HCC because of their scarcity (less than 3%) [131]. Overall, the establishment of composite scores combining PD-1 expression based on the CPS score and molecular alterations to properly predict ICI response would be a relevant strategy to explore in dedicated clinical trials.

Lastly, a recent study suggests that non-alcoholic steatohepatitis (NASH)–HCC, might be less responsive to immune checkpoint inhibitors, probably because of NASH-related aberrant T cell activation that causes tissue damage, leading to impaired immune surveillance [132]. These results need to be confirmed but with stratification of HCC patients according to aetiology (i.e., NASH-HCC or others) in immunotherapy clinical trials.

### 3.2. In Biliary Tract Cancers

#### 3.2.1. Targeted Therapies

The heterogeneity and complexity of CCA has been recently unraveled by NGS. CCAs are now recognized to exhibit a very high frequency of targetable molecular alterations [85,88,92,133,134,135]. CCA development might be promoted by chronic biliary inflammation and/or cholestasis that induce activation of molecular pathways [16,73,136,137] and mutations in genes (tumor suppressor and oncogenes) or fusions are frequent in CCA [85,88,133,134,135]. Interestingly, the anatomical and histological classification of BTC can be paralleled with molecular patterns [84,138]. To summarize data from comprehensive genomic profiling data [84], among targetable alterations, *IDH* mutations and *FGFR* 2 fusions are found in iCCA, with frequencies above 10% and up to 28% and 45% respectively [87,139,140,141,142], whereas the human epidermal growth factor receptor-2 gene (*HER2*) aberrations are more frequently observed in eCCA and GBC (up to 20%) [143]. *IDH 1/2* mutations and *FGFR2* fusions (10–15%) were described as mutually exclusive, even if some reports described a coexistence of both alterations [144].

Therefore, several targeted therapies have been or are currently tested in CCA, based on their molecular profile. *FGFR2* fusion can be targeted by FGFR inhibitors, such as pemigatinib (an oral and selective inhibitor of FGFR1, 2, and 3), BGJ398, TAS-120, or derazantinib. A multicenter phase II study (FIGHT-202) evaluated pemigatinib in patients with previously treated, locally advanced or metastatic cholangiocarcinoma were reported [33]. Among the 107 patients with *FGFR2* rearrangement or fusion, ORR was 35.5% (38/107), with three patients with a complete response. Based on these results, the FDA accelerated approval of pemigatinib for the treatment of patients with previously treated, unresectable, locally advanced or metastatic cholangiocarcinoma that harbor a *FGFR2* rearrangement or fusion.

Regarding *IDH1*/2 mutations, oral inhibitors have also been developed. Ivosidenib (AG120) targets mutated *IDH1* enzyme. The phase III ClarIDHy study [34], in 185 patients with progressing CCA, and previously treated with one or two lines of therapy, showed the superiority of ivosidenib over placebo, in terms of progression-free survival (PFS) 2.7 versus 1.4 months, *p* < 0.001) and DCR (53% vs. 28%) and estimated median OS adjusted on cross-over.

Regarding *EGFR* and *HER2*, an overexpression is observed, mostly in eCCA and GBC [133,145]. Consistently, considering the efficacy of EGFR inhibitors in other cancers like lung or colon cancer, EGFR inhibitors (such as cetuximab, lapatinib, panitumumab, and erlotinib) have been tested in CCA, but to date, different trials were disappointing with negative results even in RAS wild-type patients [146,147,148,149].

v-Raf murine sarcoma viral oncogene homolog B (*BRAF*) mutations are found in 4% of iCCA [85,150] and can be targeted. Some reported efficacy of the BRAF V600E inhibitor vemurafenib [151,152] or dabrafenib combined with trametinib (a MEK inhibitor) [153,154] and the basket trial ROAR described an efficacy of dabrafenib and trametinib with an ORR of 42% in heavily pretreated CCA patients [155]. Conversely, there are no active inhibitors for the most frequent *KRAS* mutations (non-G12C) in BTC.

In addition, neurotrophin receptor tyrosine kinase (*NTRK*) fusion is another very interesting target, which can be addressed with novel NTRK inhibitors. Approved by FDA and EMA in an agnostic way for cancers with *NTRK* fusion based on a basket trial, larotrectinib proved efficacy, including in CCA [156,157]. In the study evaluating larotrectinib, two patients (4%) with a CCA were included, of whom one experienced a major partial response [156]. In the pooled analysis of entrectinib trials, the only patient with cholangiocarcinoma experienced a partial response with a duration of response of 9.3 months and a PFS of 12.0 months [158].

*BRCA* mutations have also been described in CCAs (4%) [159]. Poly-ADP ribose polymerase (PARP) inhibitors showed efficiency in several tumor subtypes with germline or somatic mutations of *BRCA* or of *BRCA* associated genes. Data are lacking but small reports showed prolonged responses under PARP inhibitors in patients with a BRCA mutation [160]. The prevalence of DNA-damage repair (DDR) pathway defects in BTC has been ranges between 28.9% and 63.5%, but this wide range of frequencies depends on different methods for testing and on different definition of DDR alterations in BTC [161]. To date, the efficacy of PARP inhibitors in BTC patients with DDR gene alterations is unknown, and only few case reports have been described [161].

The PI3K/AKT pathway is also a promising pathway, which has been showed to be implicated in resistance to chemotherapy. In a phase II study, copanlisib, a PI3K inhibitor, failed to improve outcomes in combination with gemcitabine and cisplatin in first line in patients with advanced/unresectable BTC [162]. However, PFS and OS were greater in the group of patients with high PTEN expression (8.5 versus 4.6 months and 17.9 versus 7 months respectively) suggesting that PI3K inhibitors could be interesting if guided by a molecular screening.

Based on the current evidence and on these different studies, ESMO recommends routine use of NGS on tumors such as advanced cholangiocarcinoma. ESMO considers that large multigene panels can be used if they add acceptable extra-cost compared with small panels.

#### 3.2.2. Immunotherapies

Interestingly, between 5 and 10% of BTC exhibit DNA mismatch repair deficiency (dMMR) and/or MSI [163]. This phenotype is characterized by a high number of neoantigens activating antitumor T-cell response and associated with durable responses to ICIs in several solid tumors, including BTCs [164]. MSI BTC tumors are expected to respond well to ICI and pembrolizumab was recently approved by the FDA for patients with metastatic and/or unresectable dMMR or MSI solid tumors that progressed after prior therapy regardless of tumor type [165].

Moreover, tumors with a very high TMB are expected to respond favorably to ICI. In the KEYNOTE-158, response rates were significantly better in patients with a mutation load greater than 10 mutation/Mb compared to those with a lower TMB [166]. Unfortunately, data for TMB in BTC is limited. Recently 309 patients with BTC were reviewed, and TMB of ≥6 Mut/Mb was found in 19.4% of cases whereas TMB > 20 Mut/Mb was only identified in 2.9% of cases [167]. In the KEYNOTE-158 trial, none of the 63 CCA had a TMB > 10 Mut/Mb [166]. A recent retrospective study aimed at examining the performance of a universal definition of high TMB in an independent cohort of patients with solid tumors treated with ICIs. Among 57 patients with hepatobiliary tumors (BTC and HCC), only 4 (8%) had a high TMB ≥ 10 Mut/Mb; none responded to immunotherapy [168]. Conversely, in the low TMB group, six patients (12%) had an objective response. This does not mean that BTC cannot benefit from immunotherapy but it is crucial to develop strategies increasing the antigenic presentation such as combination with chemotherapy or radiation therapy or targeted therapy. It is also crucial to develop strong and efficient biomarkers beyond TMB to better select patients for immune therapies. Of note, TMB is significantly higher in eCCA (18%) and GBC (22%) in comparison with iCCA (13%) [169].

PD-L1 expression is often described as a predictive biomarker for ICI. Nevertheless, PD-L1 expression is particularly inefficient in BTC. Indeed, in the KEYNOTE-158 study, 58% of patients had PD-L1 expression >1% while only 6% of patients had a response [170]. ORR was 6.6% (4/61) and 2.9% (1/34) and median PFS was 1.9 and 2.1 months in the PD-L1-expressing and PD-L1 non-expressing subgroup respectively.

Reliable and predictive biomarkers are still to be developed for CCA since current biomarkers for immunotherapy response are imperfect.

## 4. What is Useful in Clinical Practice?

**Figure 3:**
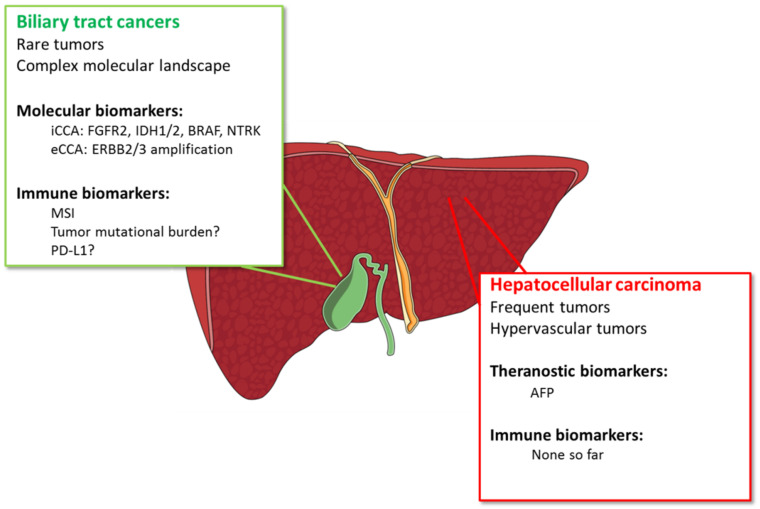
Theragnostic biomarkers used in biliary tract cancers and in hepatocellular carcinoma. Abbreviations: AFP: Alpha foeto-protein; ERBB: erb-b2 receptor tyrosine kinase; FGFR: fibroblast growth factor receptor; IDH: isocitrate dehydrogenase; MSI: microsatellite instability; NTRK: neurotrophic tyrosine receptor kinase; PD-L1: programmed death ligand-1.

HCC and CCA are two liver tumors with different prognosis and types of treatment. Contrary to HCC, CCA are rare tumors with complex molecular landscape. While efficient theragnostic biomarkers are lacking for HCC, CCA exhibit several actionable targets, which can be addressed by targeted therapies (Figure 3). Moreover, there is no validated blood or imaging biomarker to predict response to treatment in both tumors, except AFP for ramucirumab. The only validated biomarkers are tumor biomarkers such as FGFR, IDH, NTRK, and MSI, in the case of BTC.

Therefore, the use of NGS for advanced BTC patients is fundamental and should become a standard of management, since the outcome of patients with advanced BTC is poor, with a median OS of less than one year [171]. On the contrary, NGS is less valuable for HCC patients (except for research) and more data is needed to find which patients would benefit from immune therapy and antiangiogenic treatments.

In HCC, no theragnostic biomarker is validated so far that can be used in routine patient care. Indeed, several potential biomarkers have been associated with sorafenib response, such as *FGF3/FGF4* and *VEGFA* genomic amplification, overexpression of active VEGF receptors or elevated Mapk14-Atf296, but they are not validated for clinical routine. It is similar for biomarkers of response to immunotherapies, which still need to be found. Furthermore, classic markers of genomic instability such as MSI, high TMB, or POLE mutations does not seem relevant for HCC because of their scarcity (less than 3%).

On the contrary, detecting actionable targets though theragnostic biomarkers is highly valuable in BTC: (1) the therapeutic arsenal of advanced BTC is limited in second line [172] with FOLFOX chemotherapy regimen. This only therapeutic second-line validated to date in a randomized Phase III trial, showed a very modest benefit over best supportive care, with a median OS of 6 months (one month OS gain in OS) and an ORR of 5% [31] overall responses, including long-lasting responses and/or complete responses, are frequent with IDH1/2 and FGFR2 inhibitors; (2) most molecular profiling panels would also detect rare targets, such as *NTRK* fusions, interesting since NTRK inhibitors are approved regardless of the tumor type, or other rare fusion genes for which clinical trials are available; (3) besides tumors with known prevalent oncogenic driver such as gastro-intestinal stromal tumor (GIST, *c-kit* mutations) or melanoma (BRAF mutations), BTC are among the tumors with one of the highest frequency of actionable molecular alterations. Therefore, molecular screening with NGS is becoming a standard of care for BTC; (4) even if looking for *IDH1* and/or *FGFR* fusion is more relevant in iCCA, NGS allows an identification of several potential actionable targets and it is interesting to be performed regardless of primary tumor localization (e.g., *EGFR* and *HER2* in eCCA and GBC); (5) in addition to solid tissue biopsies, the place of ‘liquid biopsies’ (ctDNA), allowing repeated molecular profiling in a less invasive way, still needs to be validated in BTC. All this is consistent with the new ESMO Scale for Clinical Actionability of molecular Targets (ESCAT) classification that prioritize useful biomarkers for BTC, which have a predictive effect.

Regarding interesting theragnostic markers for immune therapies, efficient biomarkers are still lacking, and to date, only MSI/MSS (with biomolecular technics) (or dMMR/pMMR with immunohistochemistry) profile seems validated in BTC. Identification of dMMR or MSI BTC is important to propose pembrolizumab that was recently approved by the FDA for patients with metastatic and/or unresectable dMMR or MSI solid tumors that progressed after prior therapy regardless of tumor type [165]. Data are also lacking for use of TMB and/or PDL1 as predictive markers of response to immune therapies in BTC patients, and no treatment is validated so far based on these criteria. Cancer angiogenesis is a key factor for the success of immunotherapies. A crosstalk between adaptive immune cells and the cancer endothelium has been proved to be crucial for tumor immune surveillance and the success of immunotherapies. Several molecular actors have been identified such as proangiogenic molecules (FGF2, NEU1, and VEGF), soluble factors (chemokines and cytokines), immune checkpoints (PD-L1/2 and ENO-1), major histocompatibility complex (MHC 1 and 2), and adhesion molecules (selectins or integrins) [173]. Some of these actors could be of novel biomarkers in HCC and BTC and research might unveil their specific role in these diseases.

## 5. Conclusions

HCC and BTC are two diseases emerging from the liver, but with a very different clinical, pathological, and molecular profile. While several theragnostic biomarkers are validated in CCA, allowing the use of several efficient targeted therapies, no theragnostic biomarker is approved yet in HCC. Next-generation biomarkers are urgently needed to improve patients’ management and outcomes. International studies are crucial to help to identify and validate these next-generation biomarkers, and will also help to identify specify environmental differences across world regions (with different risk factors of disease). In the absence of efficient biomarkers, hypothesis-generating clinical trials are needed, with multi-omics ancillary studies, or retrospective-based ancillary studies. The next step is to perform biomarkers-based prospective therapeutic trials, based on biomarkers identified from ancillary studies.

In HCC, there is indeed still a lack of reliable biomarkers to predict response to current therapies (antiangiogenic and immune therapies) since no clear oncogenic addiction loops have been reported in HCC so far. Currently, the therapeutic management of HCC is based on the tumor extension and on patient’s characteristics (i.e., cirrhosis and performance status), leading to clinical prognostic classifications. Several scoring systems have been developed to guide therapeutic management but they are not based on molecular parameters, and no biomarkers can help to choose the best treatment for the patient with advanced diseases. Therefore, molecular biomarkers are needed to help for patients’ management.

A more detailed comprehension of intra- and intertumoral HCC heterogeneity could provide new insights into treatment resistance.

On the contrary, the last few years proved that CCA are very heterogeneous diseases in terms of genomic and oncogenic drivers and several theragnostic biomarkers are now validated for the use of targeted therapies.

In both HCC and CCA, no efficient biomarker is validated to select the better candidates to immunotherapies. Broad genomic sequencing of these tumors should be encouraged to identify homogeneous groups of patients and determinants of response to the different therapies.

## Data Availability

Not applicable.
